# Serum anti-EIF3A autoantibody as a potential diagnostic marker for hepatocellular carcinoma

**DOI:** 10.1038/s41598-019-47365-4

**Published:** 2019-07-30

**Authors:** Chang-Kyu Heo, Hai-Min Hwang, Hye-Jung Lee, Sang-Seob Kwak, Jong-Shin Yoo, Dae-Yeul Yu, Kook-Jin Lim, Soojin Lee, Eun-Wie Cho

**Affiliations:** 1 Rare Disease Research Center, Korea Research Institute of Bioscience and Biotechnology, 125 Gwahak-ro, Yuseong-gu, Daejeon 34141 South Korea; 2 College of Bioscience and Biotechnology, Chungnam National University, 99 Daehak-ro, Yuseong-gu, Daejeon 34134 South Korea; 3 Proteometech Inc., 1101 Wooree Venture Town, 466 Gangseo-ro, Gangseo-gu, Seoul 03722 South Korea; 4 Graduate Program for Nanomedical Science, Yonsei University, 50 Yonsei-ro Seodaemun-gu, Seoul, 03722 South Korea; 5 Department of Functional Genomics, University of Science and Technology, 125 Gwahak-ro, Yuseong-gu, Daejeon 34141 South Korea; 6 Biomedical Omics Group, Korea Basic Science Institute, 162 YeonGuDanji-Ro, Ochang-eup, Cheongju, Chungbuk 28119 South Korea; 7 Disease Model Research Laboratory, Korea Research Institute of Bioscience and Biotechnology, 125 Gwahak-ro, Yuseong-gu, Daejeon 34141 South Korea

**Keywords:** Hepatocellular carcinoma, Diagnostic markers

## Abstract

Tumor-associated autoantibodies are promising diagnostic biomarkers for early detection of tumors. We have screened a novel tumor-associated autoantibody in hepatocellular carcinoma (HCC) model mice. Its target antigen was identified as eukaryotic translation initiation factor 3 subunit A (EIF3A) by proteomic analysis, and the elevated expression of EIF3A in HCC tissues of tumor model mice as well as human patients was shown. Also, its existence in tumor-derived exosomes was revealed, which seem to be the cause of tumor-associated autoantibody production. To use serum anti-EIF3A autoantibody as biomarker, ELISA detecting anti-EIF3A autoantibody in human serum was performed using autoantibody-specific epitope. For the sensitive detection of serum autoantibodies its specific conformational epitopes were screened from the random cyclic peptide library, and a streptavidin antigen displaying anti-EIF3A autoantibody-specific epitope, XC90p2(-CPVRSGFPC-), was used as capture antigen. It distinguished patients with HCC (n = 102) from healthy controls (n = 0285) with a sensitivity of 79.4% and specificity of 83.5% (AUC = 0.87). Also, by simultaneously detecting with other HCC biomarkers, including alpha-fetoprotein, HCC diagnostic sensitivity improved from 79.4% to 85%. Collectively, we suggest that serum anti-EIF3A autoantibody is a useful biomarker for the diagnosis of HCC and the combinational detection of related biomarkers can enhance the accuracy of the cancer diagnosis.

## Introduction

Cancer is a major leading cause of death worldwide, which was responsible for 9.6 million deaths in 2018 according to World Health Organization (WHO) statistics^1^, implicating that nearly 1 in 6 deaths is due to cancer. Cancer outcomes can be improved by early diagnosis strategies that provide care at the most initial possible stage; thus, accurate and non-invasive *in vitro* tests are essentially necessary. Most widely used cancer biomarkers, such as carcinoembryonic antigen (CEA), alpha-fetoprotein (AFP) and prostate-specific antigen (PSA), are secreted proteins containing the signal peptides^2^. Intracellular protein antigens, which lack secretory signal sequences but released from tumor tissues^3^, are also proposed as serum biomarkers. Recently, exosomes have emerged as a new type of cancer biomarkers that includes tumor-associated antigens^4,5^. The amount of these biomarkers in the blood is, however, dependent on tumor size, which limits the use of these biomarkers for the early clinical detection of cancer. The issue of early diagnosis can be solved by tumor-associated autoantibodies, which are currently emerging early biomarkers mirroring the existence of tumor-associated antigens^6,7^. Production of tumor-associated autoantibodies by the immune system in response to tumor-associated autoantigens biologically amplifies the detectable signal of the corresponding antigens. Importantly, tumor-associated autoantibody biomarkers can be detected at the early stages of tumorigenesis, before the development of clinical symptoms^6,8^. Moreover, the long half-life of antibodies makes them as stable *in vitro* test-based biomarkers^6^. The development of an autoantibody diagnostic test also does not require expensive or complicated detection technologies, because quantification platforms are already in common clinical use^6,9^. With these advantages, a blood test measuring a panel of seven tumor-associated autoantibodies in serum, EarlyCDT-Lung test, has been developed for early diagnosis of lung cancer and it is now commercially available after extensive validation tests^10,11^.

Liver cancer has the second highest worldwide cancer mortality rate, with limited therapeutic options^12^. The most common primary hepatic malignancy is hepatocellular carcinoma (HCC), and it is predicted by a gold standard diagnostic biomarker AFP, although the lack of sensitivity^13,14^ has challenged its use. There have been continuous efforts for the discovery of HCC-associated autoantibody biomarkers^15,16^ to use as early diagnostic biomarkers, and several tumor-associated autoantibodies were recently suggested as an early predictor of HCC before diagnosis^17^. The multiplex detection of tumor-associated biomarkers also can enhance the accuracy of cancer diagnosis, which encourages further investigation of reliable autoantibody biomarkers.

HBV-encoded X protein (HBx) is known to play a pivotal role in the pathogenesis of viral-induced HCC^18,19^. The HBx transgenic (HBx-Tg) mouse was developed for the study of HCC and showed 86% incidence of the tumor with characteristics similar to human HCC^19–27^. Autoantibodies associated with human cancer can also be induced in mouse models of human cancer, similar to those occurring in human patients^8^. On the assumption that tumor-associated autoantibodies are present in the liver cancer model mouse, we prepared a B-cell hybridoma pool by fusing spleen B cells obtained from ten HBx-Tg mice bearing HCC with myeloma cells, and have screened tumor-associated autoantibodies as tumor biomarkers. In this study, we report one of these tumor-associated autoantibodies, anti-eukaryotic translation initiation factor 3 subunit A (EIF3A) autoantibody, designated as XC90, and its potential application as a diagnostic biomarker of human HCC.

## Results

### Anti-EIF3A autoantibody is identified in HBx-Tg mice

**Figure 1:**
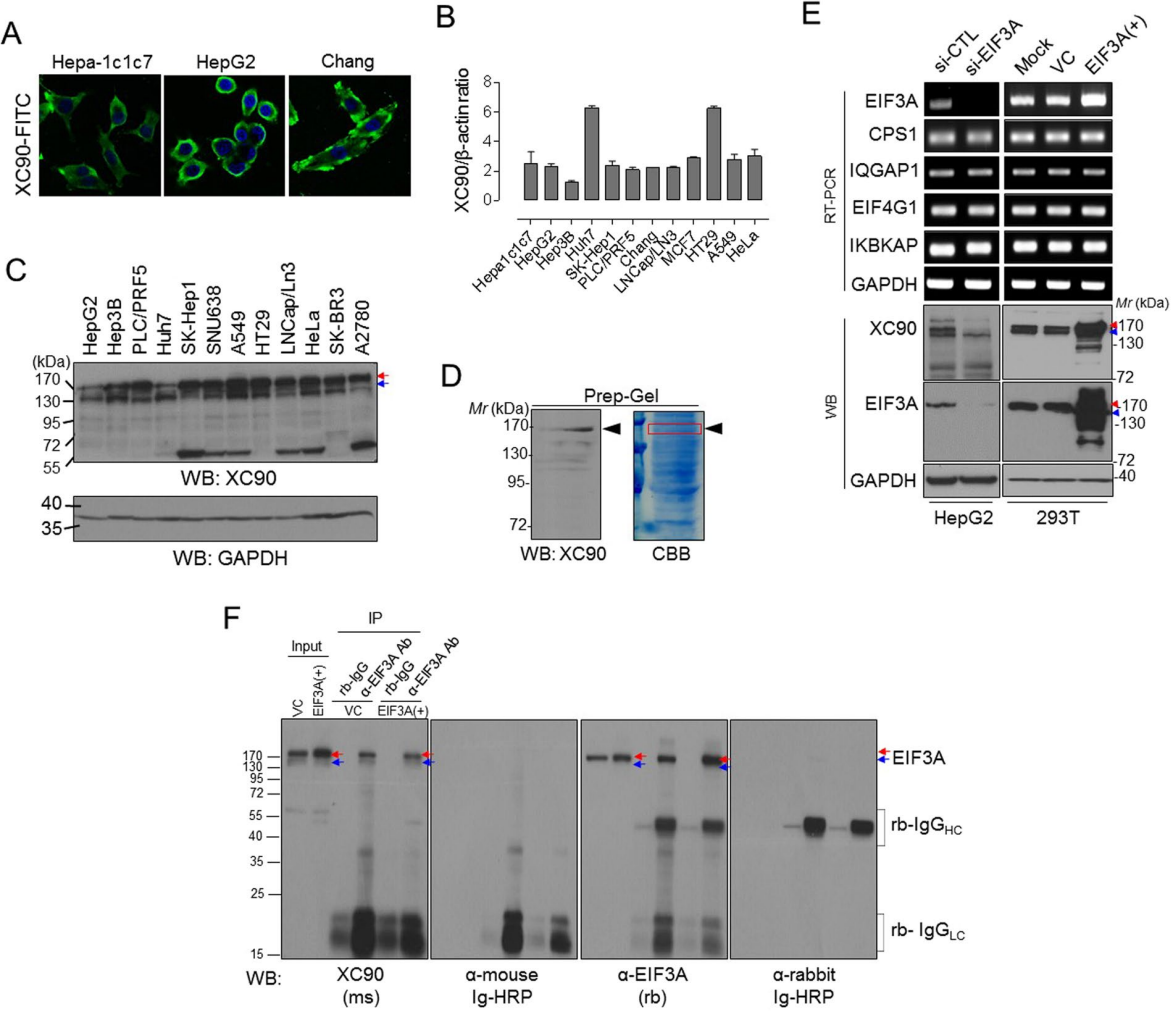
Tumor-associated autoantibody to EIF3A was identified in the human HCC model HBx-transgenic mouse. (**A**) Immunofluorescent staining of hepatoma cell lines (Hepa-1c1c7, HepG2, and Chang) with XC90 antibody. (**B**) Flow cytometric analysis of intracellularly-stained tumor cells with XC90 antibody. Binding of XC90 antibody or anti-β-actin antibody was quantified by mean fluorescence value and their relative binding ratio was plotted. (**C**) Expression of XC90 antigen in various tumor cell lines shown by Western blotting. GAPDH was served as an internal control. Arrows indicate the XC90 antigen. (**D**) Preparative 10% SDS-PAGE to isolate XC90 antigen and in-gel digestion for the mass spectrometric protein identification. The protein band containing XC90 antigen was excised (indicated by the arrow) and in-gel digested. Proteins identified by mass spectrometric analysis were listed in Supplementary Table S1. (**E**) The validation of identified proteins using EIF3A-knockdown or overexpressed cells. Cells were analyzed by RT-PCR or Western blotting. (**F**) Immunoprecipitation analysis for the verification of XC90 antigen as EIF3A. Red and blue arrows indicate EIF3A. Immunoglobulin-related proteins were indicated also, which were detected by anti-immunoglobulin secondary antibody.

To identify novel tumor-associated autoantibody biomarkers, we have individually purified antibodies produced by B-cell hybridoma clones derived from HBx-Tg mice and applied them to human liver cancer cells for immunofluorescence analysis^28^. Among these, we had identified an autoantibody named as XC90, which reacted with human HCC HepG2 cells as well as mouse hepatoma Hepa-1c1c7 cells (Fig. 1A). Fluorescence-activated cell sorting (FACS) analysis showed that XC90 antibody reacted with various hepatoma as well as non-hepatoma cancer cells (Fig. 1B). XC90 antibody, of which isotype was determined as IgM, was purified using protein L agarose (data not shown) and used for characterization. To identify the specific antigen against XC90 autoantibody, we analyzed its reactivity to cancer cell lysates by Western blotting and confirmed that it reacts with an antigen of approximately 150 kDa molecular weight (Fig. 1C). We then performed mass spectrometry (MS) analysis of protein band containing XC90 antigen (Fig. 1D, Supplementary Fig. S1), and selected five candidate proteins with a molecular weight of 150–170 kDa for further validation (protein candidates in the gray box in Supplementary Table S1). CPS1, which showed most high protein score, was examined whether it is XC90 antigen (Supplementary Fig. S2). Knockdown of CPS1 did not affect the expression of XC90 antigen. However, the change of EIF3A expression influenced on the expression of XC90 antigen; Knockdown of EIF3A inhibited the expression of XC90 antigen, and the overexpression of EIF3A increased the levels of XC90 antigen (Fig. 1E). In these conditions, the expressions of other candidate genes (CPS1, IQGAP1, EIF4G1, and IKBKAP) were not influenced. To further verify the XC90 antigen as EIF3A, EIF3A was immunoprecipitated with specific antibody commercially available and probed using XC90 antibody. As shown in Fig. 1F, EIF3A was specifically detected with XC90 antibody, and we concluded that the XC90 antigen is EIF3A, which is recently spotlighted as a novel anticancer drug target^29^.

### EIF3A expression in HCC tissue is significantly higher than that of normal controls in tumor model mice as well as patients

**Figure 2:**
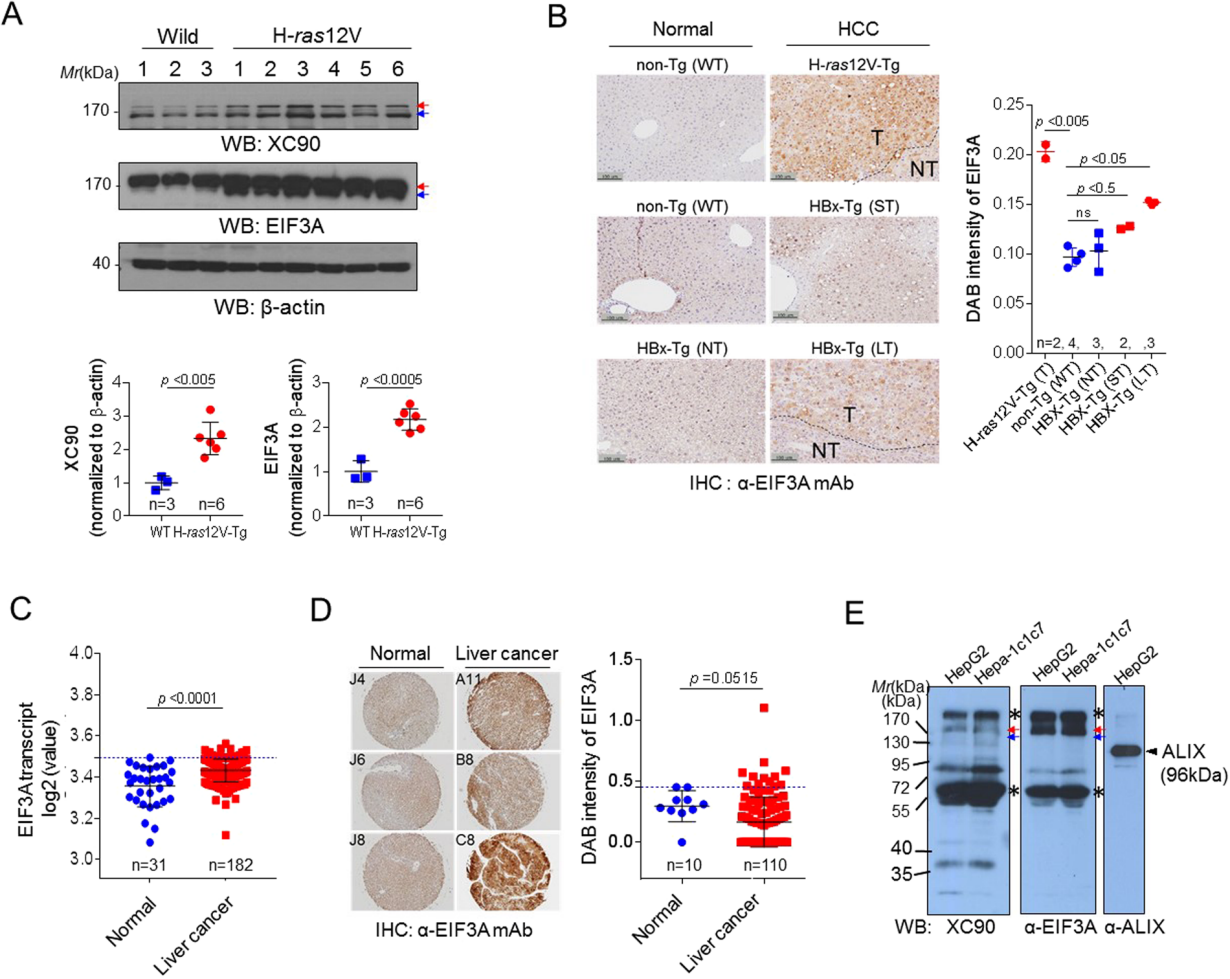
EIF3A expression was increased in HCC tissues of tumor model mice as well as patients with HCC compared to normal subjects. (**A**) The expression of EIF3A in liver tissues of H-*ras*12V-Tg mice. Blots were probed with commercial anti-EIF3A antibody and XC90. Band intensities were quantified by Image J software and the values were normalized to β-actin. (**B**) Immunohistochemical analysis of EIF3A in liver tissues of HCC model mice. Liver tissues from wild type control mice (Non-Tg: WT) were also stained. NT: non-tumor, T: tumor region, H-*ras*12V-Tg (n = 2), Non-Tg (n = 4), HBX-Tg-nonT: HBX-transgenic mouse without tumor (n = 3), HBX-Tg-ST: HBX-transgenic mouse with small tumor (n = 2), HBX-Tg-LT: HBX-transgenic mouse with large tumor (n = 3). Representative images were shown (all staining images were shown in Supplementary Fig. S3). DAB intensity of each image was quantified using Image J. (**C**) Gene expression analysis of EIF3A in human tumor tissues using GENT. (**D**) Immunohistochemical staining of human liver tissues microarray with anti-EIF3A antibody (Normal liver = 10, liver cancer = 110 cases). Representative images were shown (all staining images were shown in Supplementary Fig. S4). Statistical significance was determined by two-tailed Student’s t-test. (**E**) EIF3A in exosomes purified from hepatoma cell cultured media (HepG2 and Hepa-1c1c7 cells) analyzed by Western blotting. A well-known marker of the exosomal fraction, ALIX, was probed as a control.

EIF3A is the largest subunit of EIF3 complex, which possess RNA-binding motif for the protein synthesis initiation^29,30^. It is ubiquitously expressed at low levels in all normal adult tissues^30^. In some proliferating tissues, however, its expression is high, such as bone marrow and fetal tissues and plays essential roles in some biological processes^31–33^. Recently, reports on a relationship between EIF3A and cancer have increased, suggesting that its functions related to cancer progression^29,34^. Considering its ubiquitous expression in normal adult tissues and immune tolerance to self, antibody against EIF3A must be suppressed in a healthy body; however, autoantibody response can be induced by altered properties of self-antigen, for example, changes in the expression levels, post-translational modifications, or intracellular locations during tumorigenesis. We found that EIF3A expression was significantly increased in HCC tissues from H-*ras*12V transgenic (H-*ras*12V-Tg) mice, another model of human liver cancer^20,35^, by two-fold (Fig. 2A), compared to normal tissues when detected by a commercial antibody for EIF3A (*p* < 0.0005) as well as XC90 antibody (*p* < 0.005). Elevated EIF3A levels in HCC were also confirmed by immunohistochemical (IHC) analysis of HCC tissues from H-*ras*12V-Tg or HBx-Tg mice (Fig. 2B, Supplementary Fig. S3). Notably, H-*ras*12V-Tg mice showed highly elevated expression of EIF3A (*p* < 0.005). The expression analysis by GENT (Gene Expression across Normal and Tumor tissue, http://medicalgenome.kribb.re.kr/GENT/) also showed that EIF3A is significantly increased in human liver cancer compared to normal tissue (*p* < 0.0001; Fig. 2C). IHC analysis of a human liver cancer tissue microarray confirmed high EIF3A expression compared to normal tissue (Fig. 2D, Supplementary Fig. S4), which indicates that EIF3A overexpression may be induced during HCC progression.

The elevated intracellular antigen must be presented to the immune system to induce a specific tumor-associated autoantibody. Chronic inflammatory responses in cancer have been thought to facilitate the release and exposure of intracellular antigens to the immune system, resulting in autoantibody production^36^. Recent studies, however, have shown that intracellular components can be actively secreted by extracellular vesicles, such as exosome, without inflammation or cell death, and the release of exosomes is known to increase with the progression of cancer. EIF3A has been already identified as an exosome component released from several cancers, including bladder, ovarian, and prostate cancer^37–40^. Therefore, we examined whether intracellular EIF3A is included in the exosomes of HCC cells. Exosomes enriched by differential ultracentrifugation of hepatoma cell-cultured media (HepG2 and Hepa-1c1c7) were analyzed by Western blotting and was stained with XC90 or anti-EIF3A antibody. Several bands including 150 kDa EIF3A were stained with both antibodies in a similar pattern. We thought these proteins bands can confer epitopes that induce tumor-associated anti-EIF3A antibody, although they are not intact but may be modified by phosphorylation or degradation (Fig. 2E).

### Specific epitopes against XC90 antibody were selected from the random cyclic peptide (-CX_7_C-) library for the detection of serum anti-EIF3A autoantibody

**Figure 3:**
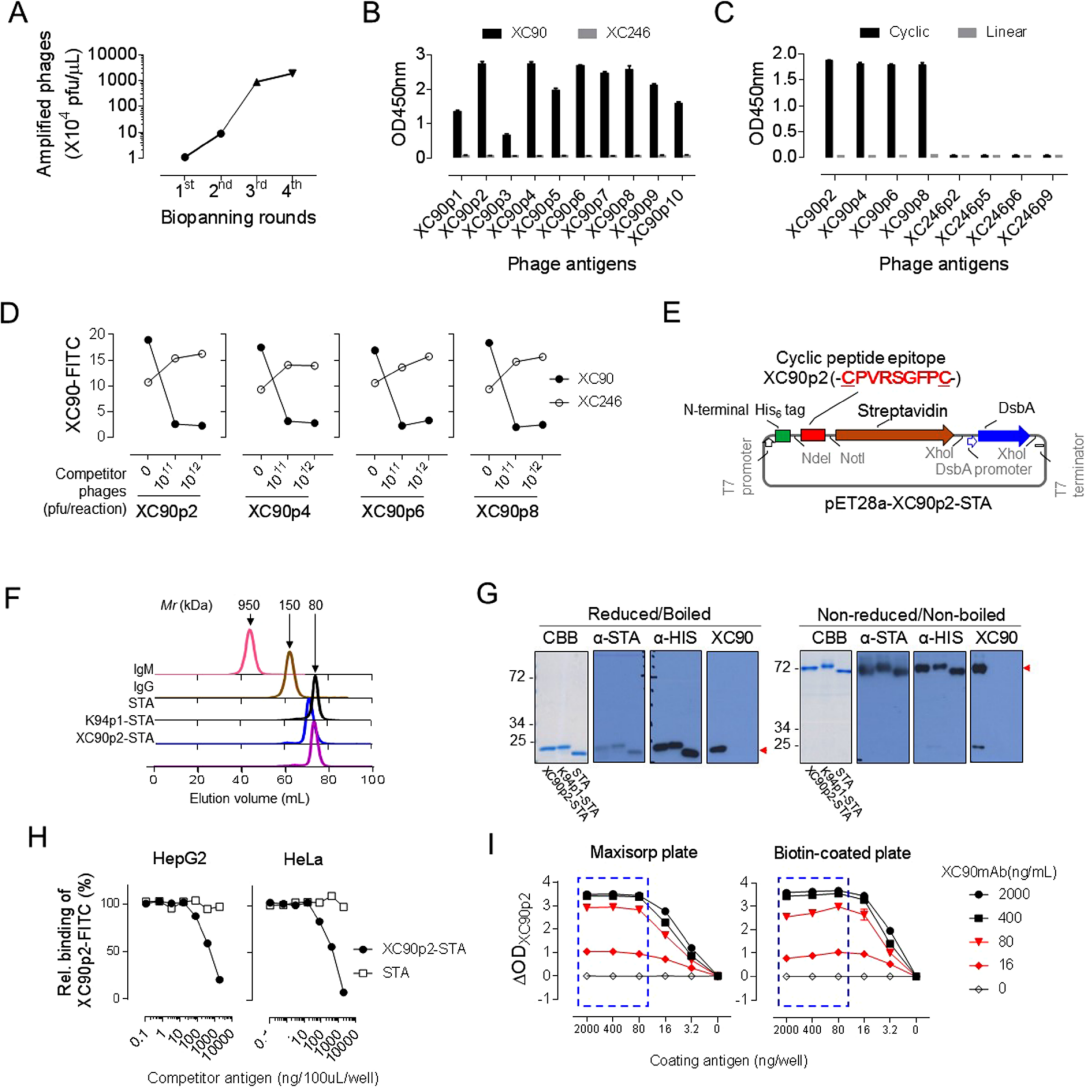
Anti-EIF3A autoantibody ELISA was set up using XC90p2-STA for specific and sensitive detection of autoantibodies in human sera. (**A**) Bio-panning with XC90 antibody against random cyclic hepta-peptide phage-displayed library. (**B**) Phage ELISA to confirm the specific binding of selected mimotopes against XC90. XC246, another autoantibody was compared as a non-related control. (**C**) The disappearance of antibody specific binding by linearizing cyclic peptide mimotopes. The phage-displayed mimotope peptides, either reduced (Linear) or non-reduced (Cyclic) were tested for their reactivity to XC90 antibody. The XC246 antibody-specific mimotope phages, including XC246p2, were used as control antigens; their epitope peptide sequences were: XC246p2:-CSSQWLPFC-; XC246p5:-CNQVAYPWC-; XC246p6: -CFSALYPWC-; and XC246p9:- CTSVFLPHC-. (**D**) Competitive FACS assay to evaluate specific binding of XC90 antibody to HepG2 cells using specific cyclic peptide mimotopes (XC90p2, XC90p4, XC90p6, XC90p8). XC246 antibody was used as control. (**E**) Expression vector construction of XC90p2 cyclic peptide-fused STA (XC90p2-STA). (**F**) Size-exclusion chromatography elution profile of XC90p2-STA. The profile plots absorption at 280 nm versus elution volume (mL). Elution profile was compared with those of other proteins [mature streptavidin without epitope (STA), K94p1 epitope-fused streptavidin (K94p1-STA), mouse IgM, and IgG] to confirm tetrameric conformation of STA with molecular weight about 80 kDa. (**G**) SDS-PAGE and Western blot analysis of XC90p2-STA to confirm the oligomeric status of XC90p2-STA. The monomer forms of STA antigens were confirmed by the analysis of reduced as well as boiled antigens. The tetrameric forms were also confirmed by analyzing non-reduced and non-boiled antigens [Coomassie Brilliant Blue staining (CBB); Immunostaining with anti-streptavidin antibody (α-STA), anti-His antibody (α-HIS) or XC90 autoantibody (XC90)]. (**H**) Competitive FACS analysis of intracellular stained cells with XC90 autoantibody. Fixed/permeabilized cells (HepG2 or HeLa) were stained with XC90 antibody pre-incubated with XC90-STA or STA at the indicated amounts and analyzed. (**I**) XC90p2 epitope ELISA with XC90p2-STA antigen. Antigen was coated as indicated amount and detected with gradually diluted XC90 antibody.

We have expected that EIF3A-specific autoantibodies would occur in human HCC patients as in tumor model mouse which showed similar characteristics to human HCC^18–27^. It is also well known that the antibody-generating epitopes usually have distinct conformations, and their structural features are conserved in mice as well as in humans. Considering these facts, we had screened the conformational epitopes against XC90 autoantibody from the cyclic peptide library for detection of serum anti-EIF3A autoantibody as in previous studies^41,42^. Biopanning of peptide library composed of 10^11^ cyclic peptides was repeated four times against XC90 autoantibody (Fig. 3A), and phages displaying ten different epitope sequences were selected (Table 1). The selected epitope display phages were reactive to XC90 but not to another autoantibody XC246 in ELISA, showing their specificity to XC90 autoantibody (Fig. 3B). Among the selected peptide epitopes, XC90p2 sequence was highly reactive (Fig. 3B, Table 1), and the reactive epitope sequences were characterized by a consensus sequence of the PxRSGxx type (P: proline; R: arginine; S: serine; G: glycine; x: diverse amino acids except for cysteine). The binding activities of selected phages were disappeared when the cyclic structures were linearized by reducing the disulfide bonds (Fig. 3C), indicating that the conformations of these epitopes are essential for their specific binding to XC90 antibody. We also examined whether the selected epitopes effectively mimic the endogenous antigenic structures by competitive FACS analysis (Fig. 3D). XC90 antibody bound specifically to HepG2 cells; however, the addition of phages displaying XC90 specific epitopes inhibited the specific binding of XC90 antibody to an almost undetectable level, but not that of XC246 antibody.

**Table 1:** Peptide sequences of mimotope against anti-EIF3A autoantibody XC90.

Phage antigen	Epitope sequence (-CXXXXXXXC-)^a^							OD_XC90_^b^
XC90p2	P	V	R	S	G	F	P	2.738
XC90p9	P	A	R	S	G	Y	P	2.126
XC90p7	P	A	R	T	S	W	P	2.475
XC90p8	P	P	R	T	G	F	Q	2.574
XC90p6	P	A	R	H	S	G	F	2.702
XC90p4	P	S	R	H	S	G	W	2.737
XC90p5	P	S	R	H	S	G	Y	1.985
XC90p10	F	P	F	P	S	S	L	1.594
XC90p1	F	P	F	P	S	S	L	1.376
XC90p3	L	P	W	P	S	S	L	0.683

^a^C: cysteine residues forming disulfide bond of cyclic peptide epitope; X: diverse amino acids except cysteine.

^b^OD_XC90_: Average activity of each phage antigen against XC90 mAb measured by enzyme-linked immunosorbent assay (ELISA), which is shown in Fig. 3B.

### Human serum anti-EIF3A autoantibody ELISA distinguished HCC patients from normal subjects

M13 phages displaying conformational peptides are useful for epitope screening. Also, an examination of their specific bindings to autoantibodies in clinical samples is possible for small scale test, as in previous studies^41,42^. However, because preparing phages not suitable for large production, epitopes were expressed in a prokaryotic system which can facilitate the formation of intracellular disulfide bonds as epitope-streptavidin (STA) fusion protein (Fig. 3E). Expression of epitope display STA tetramer was confirmed by size-exclusion chromatography and non-reducing SDS-PAGE (Fig. 3F,G). Specific binding of XC90p2 epitope-fused streptavidin against XC90 antibody was also confirmed (Fig. 3G), and its inherent epitope-mimetic ability was examined by competitive FACS (Fig. 3H).

To set up ELISA for anti-EIF3A autoantibody in human serum, conditions of ELISA were optimized. First, Maxisorp plates and biotin-coated plates were tested to select the appropriate solid phase for antigen coating (Fig. 3I). Binding of XC90 antibody to the XC90p2-STA was saturated at the coating antigen amount about 80 ng/well for both types of plates. Unexpectedly, antibody binding in biotin-coated plates was decreased slightly when the amount of coating antigen was over 80 ng/well, which was particularly noticeable at low concentrations of primary antibody (80 or 16 ng/mL). Therefore, Maxisorp plate was selected as a solid phase for coating the epitope-display STA antigen, which should detect autoantibodies at low concentrations more reliably. Human serum samples were also pretreated with albumin-removal resin and diluted to 50-fold in protein-free blocking buffer (PFBB) because serum albumin, a common source of reducing potential in blood^43^, can disturb the disulfide bonds of cyclic peptide epitopes.

**Figure 4:**
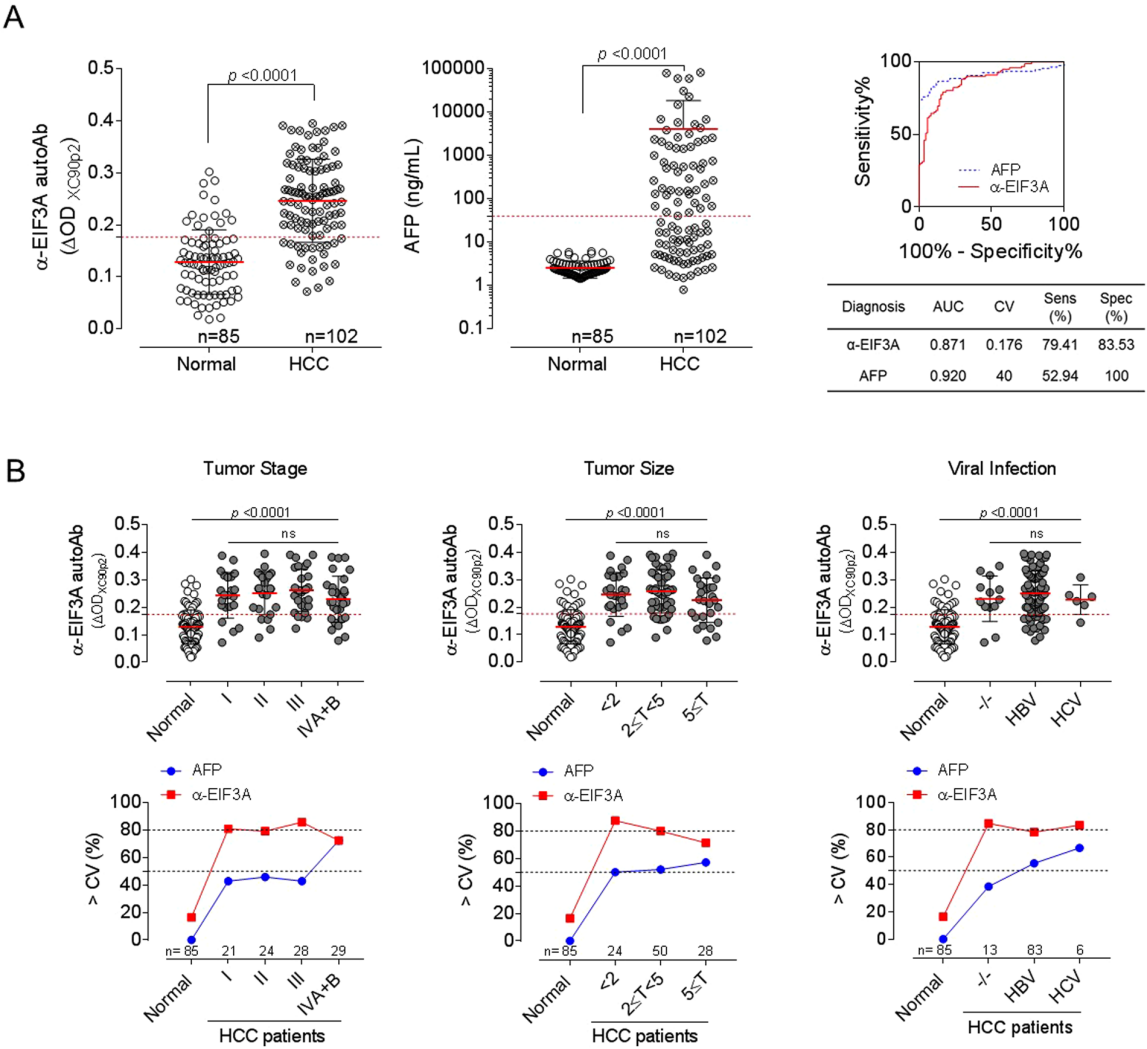
Human serum anti-EIF3A ELISA using XC90p2-STA effectively distinguished HCC patients from non-HCC subjects. (**A**) Anti-EIF3A autoantibody ELISA using XC90p2-STA in HCC patient’s sera as well as normal subjects. The specific binding of serum autoantibody to XC90p2 epitope was described as the difference in detection values between XC90p2-STA reaction and that of STA reaction (ΔOD_XC90p2_). AFP was detected also in the same sample set. Non-HCC (n = 85), HCC (n = 102). ROC curve analysis was performed to evaluate the diagnostic efficiency of each biomarker. All experiments were performed in duplicate and repeated a least three times. (**B**) Anti-EIF3A response related to TNM stage, tumor size or viral infection. The top panels show the anti-XC90p2 response (ΔOD_XC90p2_) of each sample. The lower panels show the percentages of each group over cutoff value (CV). The clinicopathological features, including the above results, are described in detail in Table 2.

ELISA for serum anti-EIF3A autoantibody was performed using these conditions, and the reactivity was described as the difference in serum antibody binding to XC90p2-STA and STA (ΔOD_XC90p2_). HCC patient sera showed significantly high response against XC90p2 compared to normal controls (Fig. 4A; AUC: 0.871, 95% CI: 0.8219–0.9218, *p* < 0.0001). When the cutoff value was 0.176, the specificity of anti-EIF3A autoantibody ELISA was 83.53% and the sensitivity was 79.41%. The responses to XC90p2 were also analyzed depending on the tumor-node-metastasis (TNM) stage, size, or viral infection (Fig. 4B, Table 2). Anti-EIF3A antibodies were detected in patients’ sera at all tumor stages (TNM stage I to IV) and sizes, even at initial tumor stages (TNM stage I) or in tumor burden of small sizes (T < 2). HBV or HCV infection, risk factors for the development of HCC, did not influence the autoantibody levels compared to patients without infection. Autoantibody response was rather slightly decreased in patients infected with HBV or HCV than patients without infection. Influences of gender or age were also analyzed (Supplementary Fig. S5). HCC patients older than 70 and females showed somewhat decreased anti-EIF3A autoantibody levels. In all subgroups of HCC patients, about 70–80% showed anti-XC90p2 responses above the cutoff value. About 16% of normal samples showed anti-XC90p2 responses; however, the response levels of most normal samples were not high.

**Table 2:** Patient details and clinic-pathological features in validation cohort^a^.

Parameters	Patients n (%)	Anti-EIF3A autoantibody^b^		p value
		<CV, n (%)	≥CV, n (%)	
All cases	102 (100)	21 (20.6)	81 (79.4)	
Age distribution (y) 39–83				0.5717
<55	57 (55.9)	12 (11.8)	45 (44.1)	
≥55	45 (44.1)	9 (8.8)	36 (35.3)	
Gender				0.2424
Male	76 (74.5)	14 (13.7)	62 (60.8)	
Female	26 (25.5)	7 (6.9)	19 (18.6)	
Viral infection				0.6356
HBV^c^	83 (81.4)	18 (17.7)	65 (63.7)	
HCV^d^	6 (5.9)	1 (1.0)	5 (4.9)	
No infection	13 (12.7)	2 (1.9)	11 (10.8)	
Tumor size				0.3626
<2 cm	26 (25.5)	4 (3.9)	22 (21.6)	
≥2 cm, <5 cm	48 (47.0)	9 (8.8)	39 (38.2)	
≥5 cm	28 (27.5)	8 (7.8)	20 (19.6)	
Portal vein and hepatic vein invasion				0.8163
No	55 (53.9)	11 (10.8)	44 (43.1)	
Yes	47 (46.1)	10 (9.8)	37 (36.3)	
Tumor multi-nodularity				0.1307
Single	62 (60.8)	11 (10.8)	51 (50.0)	
Multiple	40 (39.2)	10 (9.8)	30 (29.4)	
TNM stage^c^				0.4155
I	22 (21.6)	4 (3.9)	18 (17.7)	
II	23 (22.5)	5 (4.9)	18 (17.6)	
III	28 (27.5)	4 (3.9)	23 (22.6)	
IVA + AB	29 (28.4)	8 (7.8)	21 (20.6)	
Serum AFP concentration: 0.8−83000 ng/mL				0.5669
<40 ng/mL	48 (47.1)	12 (11.8)	36 (35.3)	
>/40 ng/mL	54 (52.9)	9 (8.8)	45 (44.1)	

^a^HCC serum samples were provided by National Biobank of Korea (Ajou Human Bio-Resource Bank).

^b^The cutoff value (CV) for serum anti-EIF3A autoantibody is 0.176, as shown in Fig. 5A.

^c^Tumor-node-metastasis (TNM) stage of HCC patients were classified according to the modified Union for International Cancer Control (UICC) system (refs^57,58^).

HBV, hepatitis B virus; HCV, hepatitis C virus; AFP, alpha-fetoprotein.

### Combined detection anti-EIF3A autoantibody and AFP in patient sera improved the accuracy of HCC diagnosis

**Figure 5:**
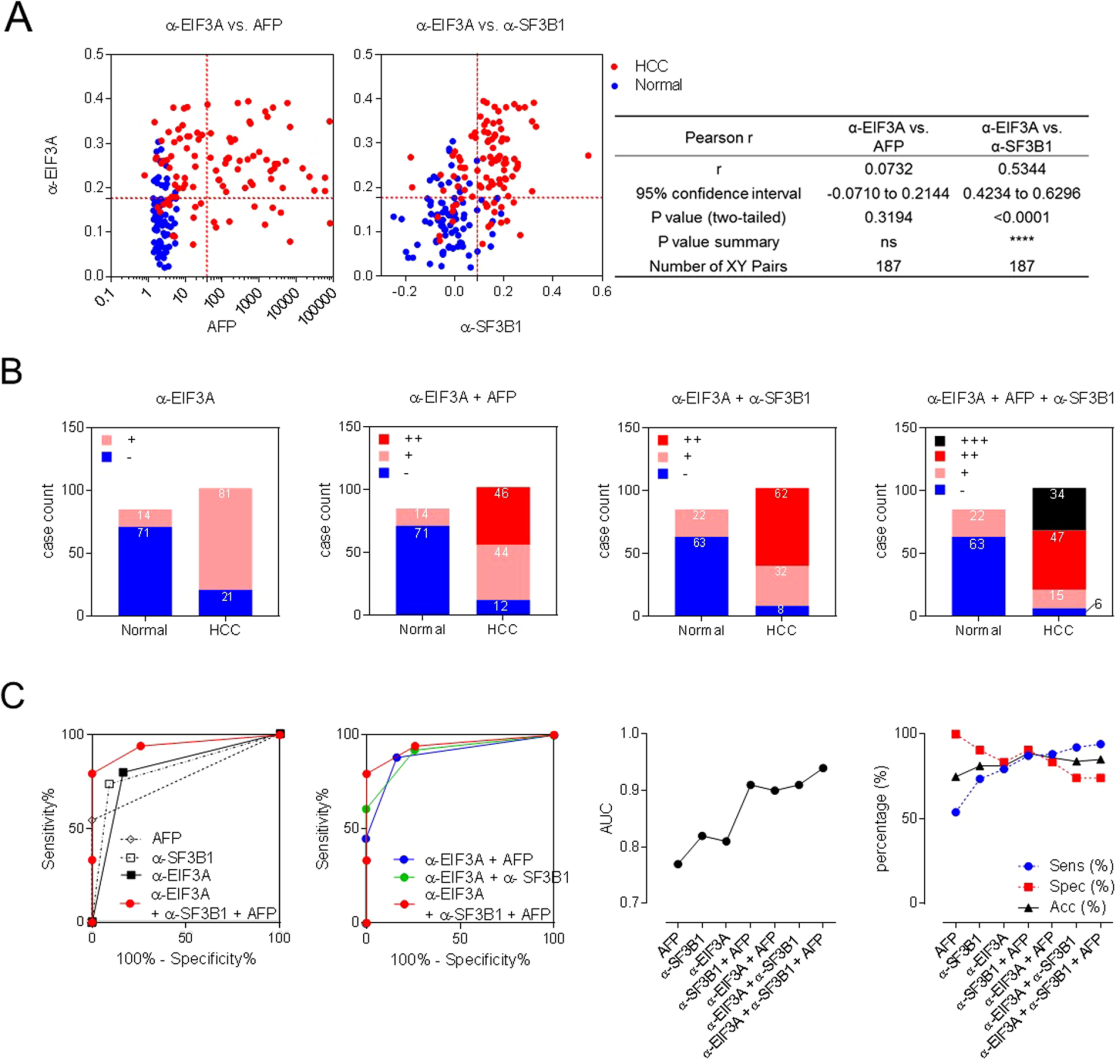
Combinational analysis of HCC biomarkers enhanced the diagnostic accuracy. (**A**) Pearson analysis of correlations between anti-EIF3A autoantibody biomarker and AFP or anti-SF3B1 autoantibody was performed. (**B**) Combinational analysis of HCC biomarkers, anti-EIF3A autoantibody, AFP, or anti-SF3B1 autoantibody. The diagnostic values of each biomarker shown in Fig. 4A (anti-EIF3A and AFP) and Fig. 5A (anti-SF3B1) were simplified as either responsive (+) or non-responsive (−) according to whether their detection values were above or below the cutoff value. Then, we analyzed the diagnostic values of each biomarker or their combination: for the combined analysis of these markers, we simply added the unified diagnostic indexes of a serum sample and designated triple negative as -, single positive as in, double positive as ++, and triple positive as +++. Numbers on plots represent the number of corresponding subjects. (**C**) ROC curve analysis of combined biomarker tests with unified index shown in Fig. 5B. AFP and anti-SF3B1 autoantibody test with unified index were shown in Supplementary Fig S6 and the results of their ROC curve analysis were also plotted (left panels). AUC values of each ROC curve analysis were plotted. Sensitivity and accuracy of each test were also plotted (right panels) and these values were shown in Table 3.

In spite of low sensitivity in the diagnosis of HCC (about 50%)^14^, AFP has been considered as the gold standard biomarker for HCC based on accumulated clinical experiences for decades. Therefore, a novel diagnostic assay for HCC can be more easily acceptable for clinical use when complementing the drawbacks of the AFP test. We performed anti-EIF3A autoantibody test and AFP test simultaneously and examined whether autoantibody test can provide additional information on HCC diagnosis. Serum AFP tests for HCC diagnosis were performed with the cutoff value of 40 ng/mL for the same sets of sera which were used to detect anti-EIF3A autoantibody biomarker. The sensitivity and specificity of AFP test were 52.94% and 100%, respectively (Fig. 4A; AUC: 0.92, 95% CI: 0.8781–0.9637, *p* < 0.0001). Pearson analysis showed no correlation between anti-EIF3A antibody and AFP biomarker (Pearson r = 0.0732, Fig. 5A left panel); however, anti-SF3B1 autoantibody, another tumor-associated autoantibody biomarker reported in our previous study^28^, showed a positive correlation with anti-EIF3A antibody (Pearson r = 0.5344), but not the same (Fig. 5A right panel). Anti-SF3B1 autoantibody also had shown no correlation with AFP in HCC diagnosis^28^. To improve the accuracy of HCC diagnosis, a multiplex biomarker analysis was performed using an index integrating the diagnostic values of each biomarker which are described by different ranges of detection values. The diagnostic values of each biomarker were simplified as either response positive (+) or negative (−), according to whether their detection values were above or below the cutoff value. Then, we analyzed the diagnostic values of each biomarker or their combinations: for the combined analysis of these markers, we simply described the unified diagnostic index as triple positive (+++), double positive (++), single positive (+), or all negative (−) (Fig. 5B,C and Table 3). As shown in Fig. 5B, a combined analysis of autoantibody biomarkers and AFP enhanced the efficacy of HCC diagnosis. The combined analysis of anti-EIF3A autoantibody and AFP (α-EIF3A + AFP) decreased the incidence of false negatives by anti-EIF3A autoantibody detection (α-EIF3A) from 21 to 12 subjects. Similarly, the combinational analysis of two autoantibody biomarkers (α-EIF3A + α-SF3B1), also decreased false negatives from 21 to 8 subjects. The best result was obtained when simultaneously analyzing three biomarkers (α-EIF3A + AFP + α-SF3B1). These results were also analyzed by ROC curve (Fig. 5C). The AUC values for each biomarker were about 0.8; however, AUC value increased up to 0.94 when three markers were simultaneously analyzed. The accuracy of biomarker testing was also increased by the combinational analysis of these three biomarkers (Fig. 5C, Table 3).

**Table 3:** Diagnostic values of serum autoantibody biomarkers and AFP.

Biomarkers	AUC^a^	Sensitivity^b^ (%)	Specificity^c^ (%)	PPV^d^ (%)	NPV^e^ (%)	ACC^f^ (%)
AFP	0.77	53.9	100.0	100.0	64.4	74.9
α-SF3B1 autoAb	0.82	73.5	90.6	90.4	74.0	81.3
α-EIF3A autoAb	0.81	79.4	83.5	85.3	77.2	81.3
α-EIF3A + AFP	0.90	88.2	83.5	86.5	85.5	86.1
α-EIF3A + α-SF3B1	0.91	92.2	74.1	81.0	88.7	84.0
α-EIF3A + α-SF3B1 + AFP	0.94	94.1	74.1	81.4	91.3	85.0

^a^AUC: Area under curve.

^b^Sensitivity, True positive rate, probability of detection = Σ True positive/Σ Condition positive.

^c^Specificity, Selectivity, True negative rate = Σ True negative/Σ Condition negative.

^d^PPV: Positive predictive value, Precision = Σ True positive/Σ Predicted condition positive.

^e^NPV: Negative predictive value = Σ True negative/Σ Predicted condition negative.

^f^ACC: Accuracy = Σ True positive + Σ True negative/Σ Total population.

## Discussion

In this study, we identified anti-EIF3A autoantibody as a novel tumor-associated autoantibody biomarker in HCC model mouse as well as in human HCC patients. We showed that the increased expression level of EIF3A and its secretion from HCC cells as an exosomal component, which may elicit the antigen-specific immune responses to amplify the humoral response against itself. We detected autoantibody biomarker in human sera using specific conformational cyclic peptide epitope against anti-EIF3A monoclonal autoantibody obtained from HCC model mouse, and also performed multiplex biomarker assay consists of anti-EIF3A autoantibody and other two HCC biomarkers, which enhanced the accuracy of HCC diagnosis.

Increment of EIF3A in cancer is interesting in the aspects of tumorigenesis because EIF3A has been highlighted as a translational regulator of a specific subset of mRNA transcripts related to cell proliferation^34^. In addition, its secretion from tumor cells as an exosomal component can deliver tumor-related translational regulator to other cells which can be influenced. Other elements of EIF3 complex as well as translational machinery such as EIF3B, EIF3C, EIF4A, EEF1A, and EEF2 were already revealed as components of tumor-associated exosomes^37–40^, which implicates exosomal delivery of translational machinery including EIF3A can be involved in tumor propagation.

During the process of exosome secretion or uptake, EIF3A can be presented to circulating immune cells, which may induce autoantibody response. Consistent with our results, recently it was suggested that exosomes harbor B cell targets in cancer as well as in autoimmune disease^44^. Antigenic epitopes on exosomes have been proven to be more accessible to B cells efficiently because they may be presented and protected from proteolysis compared with the soluble form of the protein, leading to a coordinated CD4+ and CD8+ T-cell response and consequently the secretion of autoantibodies^44,45^. Also, exosomes derived from tumor cells, which are extracellular vesicles composed of lipids bilayer and various cellular components, can act as immune-adjuvants like artificial liposome nanoparticles^46^, resulting in the immune response to be favorable to tumor-associated antigens.

However, despite immune stimulation by exosomal antigens and the increase of adaptive tumor-associated autoantibodies, their functions as targeting tumor cancer cells are not expected because of the decoy effect of exosome^44^. At present, exploring the clinical potential of serum autoantibodies is focused on its use as early diagnostic biomarkers. Early detection of tumor-associated autoantibodies prior to the clinical symptom or tumor burden growth make them useful as early diagnostic biomarker^6,8,47^. In our studies, anti-ELIF3A autoantibody biomarker was detected in about 80% of HCC patients even at initial tumor (tumor stage I) or of small tumor (<2 cm), and the detection rate is not increased further, suggesting that anti-ELIF3A autoantibody biomarker might be induced prior to the development of clinical symptoms and with a minimal small size tumor burden. Tumor-secreting exosomes, although derived from minimal tumor burden, may deliver increased EIF3A antigen and display a neo-B cell epitope that is not displayed in normal exosomes, leading to produce autoantibody. Other factors which can influence to induce autoantibodies, such as gender, age, or viral infection do not seem to affect the anti-EIF3A autoantibody response significantly. The elevation of EIF3A^48–53^ or its localization in exosome^37–39^ were also reported in several other cancers, which suggests that anti-EIF3A autoantibody biomarker may be induced in other tumors. Further studies on anti-EIF3A autoantibody as a cancer biomarker in other cancers seem to need, which may expand its use.

Because of the common nature of tumorigenesis, most of the cancer biomarkers are not tissue- or tumor-specific as a single marker. Even serum AFP, which is regarded as the “gold standard” biomarker for liver cancer, is also used as a biomarker of testicular cancer accompanying with human chorionic gonadotropin (HCG), based on their highly-elevated expression in testicular cancer patients^54^. Also, because of the heterogeneity between cancer cells within a tumor burden, no single biomarker can fully diagnose specific cancer. Therefore, it is necessary to detect several biomarkers simultaneously to diagnose a certain type of cancer and then integrate the detection values as a diagnostic value for its interpretation. The benefit of a multiplex analysis of tumor-associated autoantibodies is supported by an existing multiplex autoantibody diagnostic test for lung cancer, EarlyCDT, which includes seven autoantibodies biomarkers^11,55^. We expect that the efficiency of HCC diagnosis also would be increased by using the EIF3A autoantibody biomarker with other biomarkers. In further studies, it is possible to test our autoantibody biomarkers in this study with other autoantibody biomarkers, such as anti-fatty acid synthase (FASN) autoantibody^42^ to formulate an HCC diagnosis test.

HCC-related biomarker autoantibodies suggested in our studies target large-size antigens with a molecular weight of above 140 kDa^28,41,42^. The size of B cell epitopes, however, is sufficient with about 15–20 amino acids, and the preparation of large size recombinant proteins with native conformation is fussy. Therefore, we screened conformational peptide epitope from the cyclic peptide (-CX_7_C-) library and used as detection antigens successfully. Most of autoantibody biomarker assays have used recombinant proteins as capture antigens. Although target antigen is identified accurately and is prepared successfully, epitopes displayed by recombinant protein can be not proper to detect autoantibody sensitively, which lead autoantibody biomarker tests to be less sensitive. We think that the conformational epitopes of high binding activity can enhance the efficiency of autoantibody biomarker assay suggested until now.

In conclusion, our study provides a rationale for using anti-EIF3A autoantibody as an HCC-associated biomarker and a serum ELISA using specific conformational epitope against anti-EIF3A autoantibody. We also suggest it as an accompanying test with AFP for HCC diagnosis to enhance the diagnostic efficiency by combinational analysis. It now seems to be premature to define anti-EIF3A autoantibody as early stage cancer biomarker because of the limitation in clinical samples for retrospective or prospective studies; however, further studies on large, well-defined cohort will show its usefulness for cancer diagnosis.

## Methods

### Cell cultures and human serum samples

Hepa-c1c7 mouse hepatoma cell and various human cancer cell lines obtained from the American Type Culture Collection (Manassas, VA, USA) were cultured in DMEM or RPMI-1640 (Thermo Fisher Scientific, MA, USA) supplemented with 10% fetal bovine serum (FBS; Sigma-Aldrich, St. Louis, MO, USA) and penicillin/streptomycin (Thermo Scientific) at 37 °C in 5% CO_2_. Human HCC serum samples (n = 102) before treatment were obtained from Ajou Human Bio-Resource Bank with clinicopathological information. All samples were provided with informed consent under IRB-approved protocols. This study was approved by the Public Institutional Bioethics Committee designated by MOHW (P01-201409-BS-03). Normal serum samples of non-HBV/HCV carriers and normal serum AST/ALT levels were obtained from the Korean Red Cross. Research Blood Examination Committee exempted IRB approval for these samples. Serum samples were kept at −70 °C.

### XC90 monoclonal autoantibody

For preparing B cell hybridoma clones producing tumor-associated autoantibodies, the spleen cells from HBx-Tg mouse bearing HCC (11–13 months of age) were used^28,42^, and one of stable B-cell hybridoma clones, XC90, was analyzed in this study. The antibody isotype of XC90 was determined using isotyping kit (Thermo Scientific). XC90 antibody was purified from cultured media of XC90 B-cell hybridoma using protein L agarose (Thermo Scientific). Antibody-specific immunofluorescence staining and FACS were performed as described previously^28^.

### Western blot analysis and immunoprecipitation

For Western blot analyses, cells were lysed in NP-40 cell lysis buffer [50 mM Tris-HCl (pH 7.4), 150 mM NaCl, 1% NP-40, 1 mM PMSF, and a phosphatase inhibitor cocktail (Sigma)]. Protein concentration was determined by the Bradford assay (Bio-Rad Laboratories, Hercules, CA). Equivalent amounts of protein (20–50 μg) were analyzed by Western blotting using purified XC90 antibody or specific antibodies against EIF3A (Cell Signaling Technology, Danvers, MA, USA), ALIX (CST) or β-actin (Santa Cruz Biotechnology, Dallas, TX, USA). Band intensities were quantified by Image J software (NIH, USA) and normalized to β-actin. For the competitive Western blot assay, the primary antibody XC90 was incubated with epitope display phages (indicated pfu/100 μL) prior to probing. To immunoprecipitate EIF3A, cell lysates (500 μg) were incubated with 1 μg of anti-EIF3A antibody (CST) or rabbit IgG and protein A/G beads (Santa Cruz) for 4 hours at 4 °C. After briefly washing with phosphate-buffered saline (PBS), the immunoprecipitates were analyzed by immunoblotting with anti-EIF3A antibody or XC90 antibody. The exosomes were obtained from conditioned media of tumor cells which were 70–80% confluent and cultured for 48 h with exosome-free FBS (System Biosciences, Palo Alto, CA, USA). Exosomes were obtained by differential ultracentrifugation^56^ of the conditioned media. The exosome pellets were solubilized in NP-40 cell lysis buffer and analyzed by Western blotting.

### Identification of XC90 tumor-associated antigen

For the proteomic analysis of XC90 antigen, the target protein specific to the XC90 autoantibody was enriched by the fractionation of cell lysate using HiTrap Q HP anion exchange column (GE, Boston, MA, USA). Lysate of HeLa cells was prepared with NP40 lysis buffer, and the bound protein was eluted with a linear gradient of sodium chloride from 0 to 1 M dissolved in 10 mM sodium phosphate buffer (pH 7.4). The fractions containing the XC90 antigen were confirmed by Western blot analysis. Selected fractions were collected and their concentrate was separated by SDS-PAGE, followed by Western blot analysis or Coomassie Blue staining. The Coomassie-stained bands which shown reactivity to XC90 antibody were excised and in-gel digested with Trypsin Gold (Promega, Madison, WI, USA). After cleaned up with C18 ZipTips, tryptic peptides were subjected to nano-liquid chromatography-electrospray ionization-tandem mass spectrometry (LC/ESI-MS/MS) as described previously^28^.

### Knockdown or overexpression of EIF3A and reverse transcription polymerase chain reaction (RT-PCR)

To confirm that the XC90 antigen as EIF3A, cells were transfected with siRNA (Bioneer Corporation, Daejeon, Korea) targeting EIF3A as follows; EIF3A sense: 5′-CAGUUGAUGGCAAAUUACU(dTdT)-3′, EIF3A antisense: 5′-AGUAAUUUGUCAACU G(dTdT)-3′. The EIF3A overexpression vector was constructed by cloning the *EIF3A* gene amplified from cDNA of HepG2 cells into a pEF1α-IRES-ZsGreen1 vector (Clontech Laboratories, CA, USA). EIF3A overexpressing cells or knockdown cells were analyzed 72 h after transfection. RT-PCR was performed using primers (Bioneer) shown in Supplementary Table S2.

### Immunohistochemistry

Preparation of paraffin-embedded tissue specimens from the tumor-model mouse and immunostaining with anti-EIF3A antibody (CST, 1:200 diluted) were performed following procedures described previously^19^. The tissue microarray of human HCC (BC03119) was purchased from US Biomax (Rockville, MD, USA). The photomicrographs were captured at 200× or 400× magnification.

### Bio-panning of the cyclic peptide library

For the selection of the epitope specific to XC90 antibody, the phage display random cyclic peptide library Ph.D.-C7C™ (New England Biolabs, MA, USA) was used for panning as described previously^28^.

### Expression and purification of XC90p2 epitope-fused STA

For the preparation of XC90p2 cyclic peptide epitope display streptavidin, the oligonucleotide coding XC90p2 sequence (-CPVRSGFPC-) with *Nde*I and *Not*I restriction enzyme sites was synthesized (Bioneer) and cloned into the streptavidin-expressing pET28a(+) vector, and was transformed into *E*. *coli* strain SHuffle® T7 (New England Biolabs). Transformants were cultured, and XC90p2 epitope-fused STA was purified following procedure described previously^28^.

### ELISA

ELISA for epitope-display phage was performed as described previously^28^. To examine the conformational effect of disulfide bond of epitope on binding efficacy against target antibody, cyclic peptide display phages were treated with dithiothreitol (DTT) and iodoacetamide^42^. When using epitope-fused STA as coating antigen, Maxisorp high protein-binding capacity plates or biotin plates (Thermo Scientific) were incubated with antigens of the indicated quantities in 100 μL PBS at 4 °C overnight. To detect the reactivity of patient sera to XC90p2-STA, microwell plates were coated with XC90p2-STA in PBS at the concentration of 500 ng/100 μL. Albumin-depleted human sera (1:50 diluted in PFBB) were used as primary antibodies and horseradish peroxidase (HRP)-conjugated anti-human IgGAM antibody (Thermo Scientific; 1:2000 diluted) was used to detect serum autoantibodies^28^. AFP in serum was quantified using a commercialized assay (R&D Systems, Minneapolis, MN, USA).

### Statistical analysis

Experimental data were described as the mean ± standard deviation (SD), and a two-tailed Student’s t-test was used to access the statistical significance. Receiver operating characteristic (ROC) curves were used to assess the diagnostic accuracy of biomarkers, represented by the area under the curve (AUC), using Prism 7 software (GraphPad Software, La Jolla, CA, USA). The ideal cutoff points for discriminating cancer patients from normal were assessed as the maximum sum of sensitivity and specificity.

## Supplementary information

Supplementary Information
