# Supplementary material for: Serum anti-EIF3A autoantibody as a potential diagnostic marker for hepatocellular carcinoma
Source: Sci Rep. 2019 Jul 30;9:11059. doi: 10.1038/s41598-019-47365-4 (PMC6667438; doi:10.1038/s41598-019-47365-4)
Supplement: Supplementary file 1 — Supplementary Information [file 41598_2019_47365_MOESM1_ESM.pdf]

Supplementary Information

Serum anti-EIF3A autoantibody as a potential diagnostic marker for hepatocellular carcinoma

Chang-Kyu Heo[1,2], Hai-Min Hwang[1,2], Hye-Jung Lee[3,4], Sang-Seob Kwak[1,5], Jong-Shin Yoo[6], Dae-Yeul Yu[7], Kook-Jin Lim[3], Soojin Lee[2]*, Eun-Wie Cho[1,5]*

[1]Rare Disease Research Center, Korea Research Institute of Bioscience and Biotechnology, Daejeon, South Korea, [2]College of Bioscience and Biotechnology, Chungnam National University, Daejeon, Korea, [3]Proteometech Inc., Seoul, South Korea, [4]Graduate Program for Nanomedical Science, Yonsei University, Seoul, South Korea, [5]Department of Functional Genomics, University of Science and Technology, Daejeon, South Korea, [6]Biomedical Omics Group, Korea Basic Science Institute, Cheongju, Chungbuk, South Korea, [7]Disease Model Research Laboratory, Korea Research Institute of Bioscience and Biotechnology, Daejeon, South Korea

***Correspondence:**

Soojin Lee

Telephone: +82-42-821-6414; Fax: +82-42-821-8831; Email: leesoojin@cnu.ac.kr

Eun-Wie Cho

Telephone: +82-42-860-4155; Fax: +82-42-869-8139; Email: ewcho@kribb.re.kr

Supplementary tables S1-S2

Supplementary figures S1-S6

Supplementary table S1. Mass spectrometric analysis of proteins in XC90 antigen-enriched protein band

Accession Number	Gene Symbol	Gene Name	Mass	Score	Matches	Sequences	emPAI
IPI00296337	PRKDC	Isoform 1 of DNA-dependent protein kinase catalytic subunit	468,788	76	1(1)	1(1)	0.01
IPI00026781	FASN	Fatty acid synthase	273,254	57	1(1)	1(1)	0.01
IPI00289831	PTPRS	Isoform PTPS of Receptor-type tyrosine-protein phosphatase S	216,958	52	1(1)	1(1)	0.02
IPI00107831	PTPRF	Isoform 1 of Receptor-type tyrosine-protein phosphatase F	212,744	50	1(1)	1(1)	0.02
IPI00011062	CPS1	Isoform 1 of Carbamoyl-phosphate synthase [ammonia], mitochondrial	164,835	2411	59(59)	28(28)	1.42
IPI00009342	IQGAP1	Ras GTPase-activating-like protein IQGAP1	189,134	264	4(4)	3(3)	0.06
IPI00386533	EIF4G1	Isoform E of Eukaryotic translation initiation factor 4 gamma 1	154,837	220	5(5)	4(4)	0.1
**IPI00029012**	**EIF3A**	**Eukaryotic translation initiation factor 3 subunit A**	**166,468**	**156**	**4(4)**	**3(3)**	**0.07**
IPI00293735	IKBKAP	Elongator complex protein 1	150,159	136	4(4)	3(3)	0.08
IPI00328268	EIF4G3	EIF4G3 protein	146,779	86	2(2)	1(1)	0.03
IPI00026089	SF3B1	Splicing factor 3B subunit 1	145,738	44	1(1)	1(1)	0.03
IPI00031023	FLII	Protein flightless-1 homolog	144,659	73	1(1)	1(1)	0.03
IPI00004534	PFAS	Phosphoribosylformylglycinamidine synthase	144,633	46	1(1)	1(1)	0.03
IPI00644127	IARS	Isoleucyl-tRNA synthetase, cytoplasmic	144,406	153	3(3)	2(2)	0.03
IPI00291939	SMC1A	Structural maintenance of chromosomes protein 1A	143,144	161	2(2)	1(1)	0.05
IPI00029485	DCTN1	Isoform p150 of Dynactin subunit 1	141,607	69	1(1)	1(1)	0.03
IPI00022228	HDLBP	Vigilin	141,368	144	4(4)	4(4)	0.03
IPI00852685	DIAPH1	Isoform 1 of Protein diaphanous homolog 1	141,258	121	2(2)	2(2)	0.11
IPI00414819	SKIV2L	Helicase SKI2W	137,712	42	1(1)	1(1)	0.05
IPI00300371	SF3B3	Isoform 1 of Splicing factor 3B subunit 3	135,492	56	1(1)	1(1)	0.03
IPI00465128	BAT3	Isoform 1 of Large proline-rich protein BAT3	119,334	119	2(2)	2(2)	0.03
IPI00000877	HYOU1	Hypoxia up-regulated protein 1	111,266	322	7(7)	5(5)	0.22
IPI00022744	CSE1L	Isoform 1 of Exportin-2	110,346	51	1(1)	1(1)	0.03
IPI00218628	ITGA2B	Isoform 2 of Integrin alpha-IIb	109,505	48	1(1)	1(1)	0.03
IPI00100787	RGPD8	Isoform 2 of RANBP2-like and GRIP domain-containing protein 5/6	103,269	42	1(1)	1(1)	0.04
IPI00382470	HSP90AA1	Isoform 2 of Heat shock protein HSP 90-alpha	98,099	54	1(1)	1(1)	0.04
IPI00179953	NASP	Isoform 1 of Nuclear autoantigenic sperm protein	85,186	511	12(12)	9(9)	0.47
IPI00414676	HSP90AB1	Heat shock protein HSP 90-beta	83,212	114	2(2)	1(1)	0.04
IPI00654628	ANKHD1	cDNA FLJ20288 fis, clone HEP04414 (Fragment)	73,570	96	1(1)	1(1)	0.05
IPI00299116	PODXL	Podocalyxin-like protein 1 precursor	55,536	43	1(1)	1(1)	0.07
IPI00021439	ACTB	Actin, cytoplasmic 1	41,710	53	1(1)	1(1)	0.09
IPI00166768	TUBA1C	TUBA1C protein	36,719	148	4(4)	3(3)	0.34

Supplementary table S2. RT-PCR primer sequences

Gene name	Primer sequences(5’→3’)	
	Foward	Reverse
Eukaryotic translation initiation factor 3 subunit A (EIF3A)	5′-ATGCCGGCCTATTTTCAGAG-3’	5′-TTTTTCCTCTGCCATTTTCAAAT-3’
Isoform 1 of Carbamoyl-phosphate synthase [ammonia], mitochondrial (CPS1)	5′-CTATATCAGCAGATGGTAGACA-3’	5′-AACCTTACTTCCAAGTTATTCC-3’
Ras GTPase-activating-like protein IQGAP1 (IQGAP1)	5′-GGAGCACAATGATCCAATCC-3’	5′-ATGGTTCGAGCATCCATTTC-3’
Isoform E of Eukaryotic translation initiation factor 4 gamma 1 (EIF4G1)	5′-CTGTGTGACGAGCAGAAGGA-3’	5′-CCCAACTGTAGAAGGCATCC-3’
Ikappa B kinase complex-associated protein; Elongator complex protein 1 (IKBKAP)	5′-GGTTCACGGATTGTCACTGTT-3’	5′-ACATAAGTTTGTCCAACCACTTC-3’
GAPDH	5′-TGATGACATCAAGAAGGTGGTGAAG-3’	5′-TCCTTGGAGGCCATGTGGGCCAT-3’

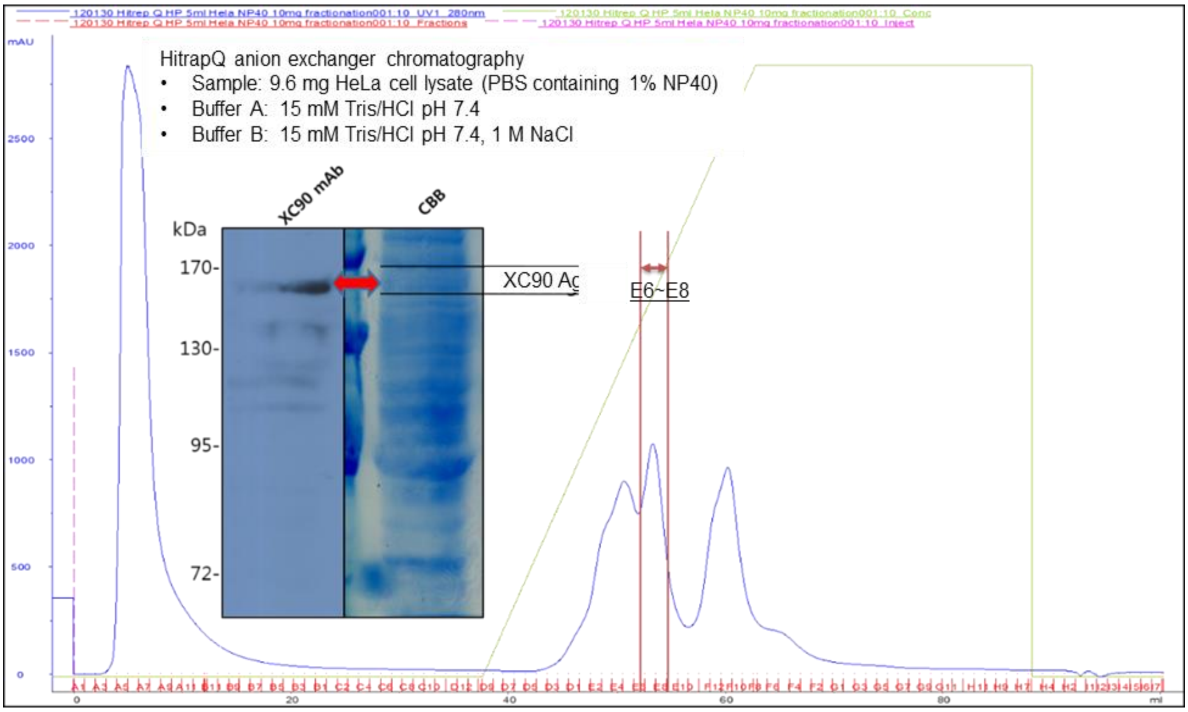

Supplementary figure S1. Enrichment of XC90 antigen by fractionation of HeLa cell lysate using anion exchange chromatography. HeLa cell lysates were fractionated using Hitrap Q anion-exchange chromatography. We pooled XC90 antigen-enriched fractions (E6~E8) that were confirmed by western blotting, and its concentrate was subjected to preparative 10% sodium dodecyl sulfate-polyacrylamide gel electrophoresis (SDS-PAGE). Then, the protein band corresponding to the XC90 antigen, confirmed by western blotting, was excised. In-gel enzymatic digestion of proteins within the excised gel followed by MS analysis identified various protein candidates (Supplementary table S1).

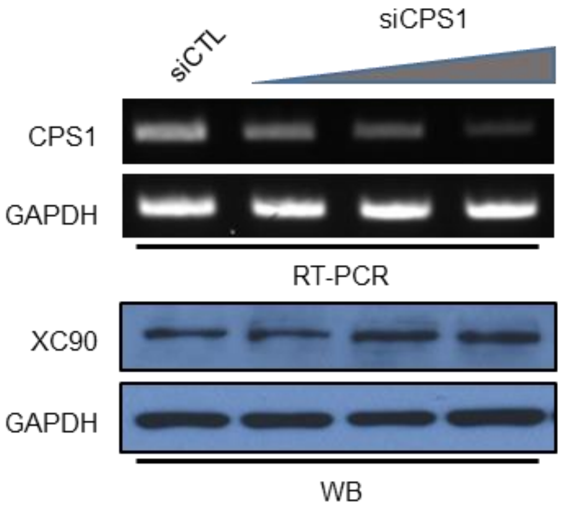

Supplementary figure S2. Validation of candidate proteins identified by mass spectrometric analysis. Knockdown of EIF3A suppressed XC90 antigen expression as shown in Fig 1E. However, suppression of CPS1, another candidate protein for XC90 antigen, showed no influence on the expression of XC90 antigen. Knockdown of CPS1 was performed by siRNA transfection and following analysis were performed as described in Methods.

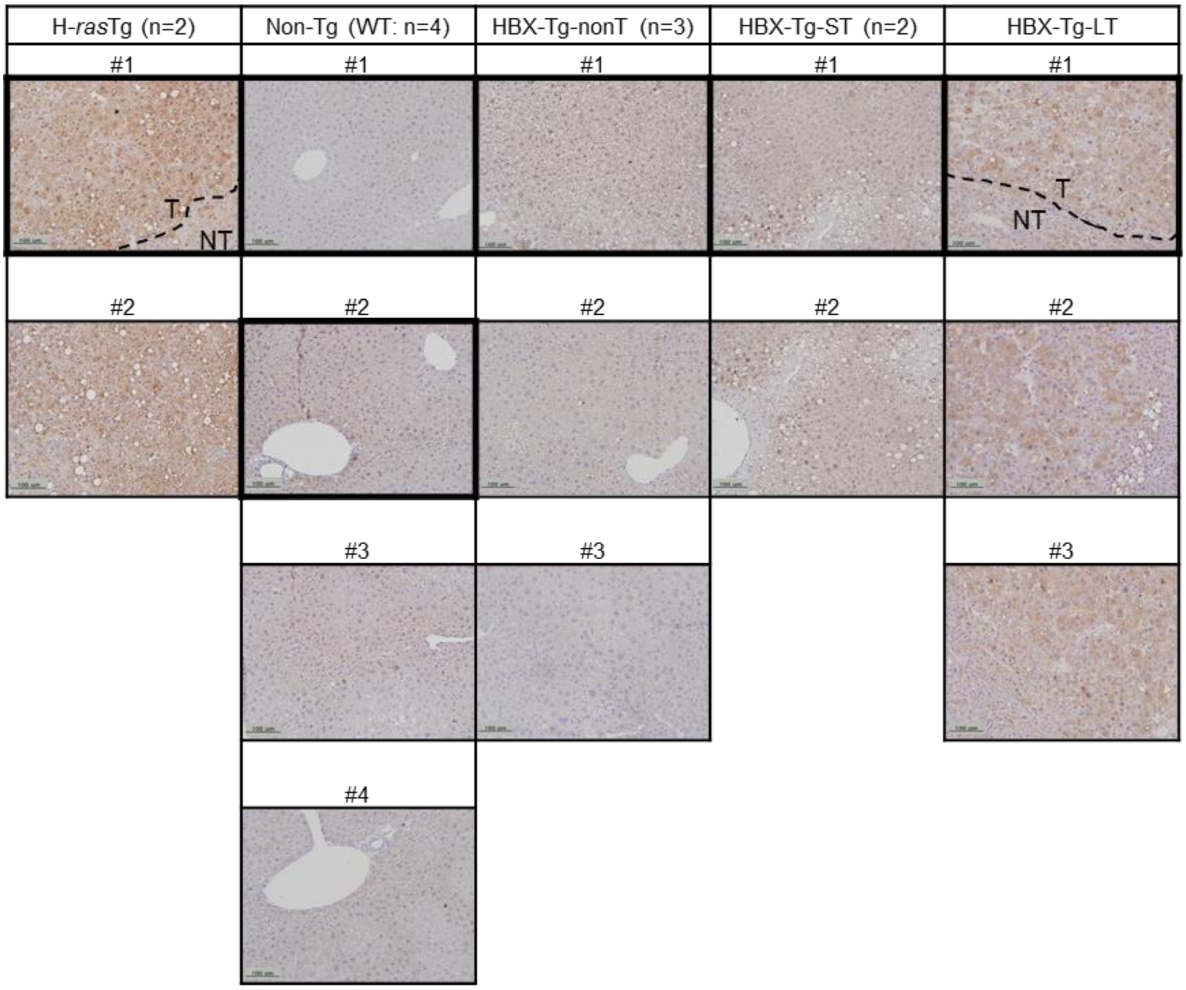

Supplementary figure S3. Immunohistochemical staining with anti-EIF3A antibody in liver tissues of HCC model mice (H-*ras*12V-Tg, HBx-Tg). Liver tissues of wild type control mice (Non-Tg: WT) were also stained. T: tumor, NT: non-tumor region, HBX-Tg-nonT: HBX-transgenic mouse without tumor, ST: small tumor, LT: large tumor. Representative images were shown in Fig. 2B and DAB intensity of each image was quantified and plotted.

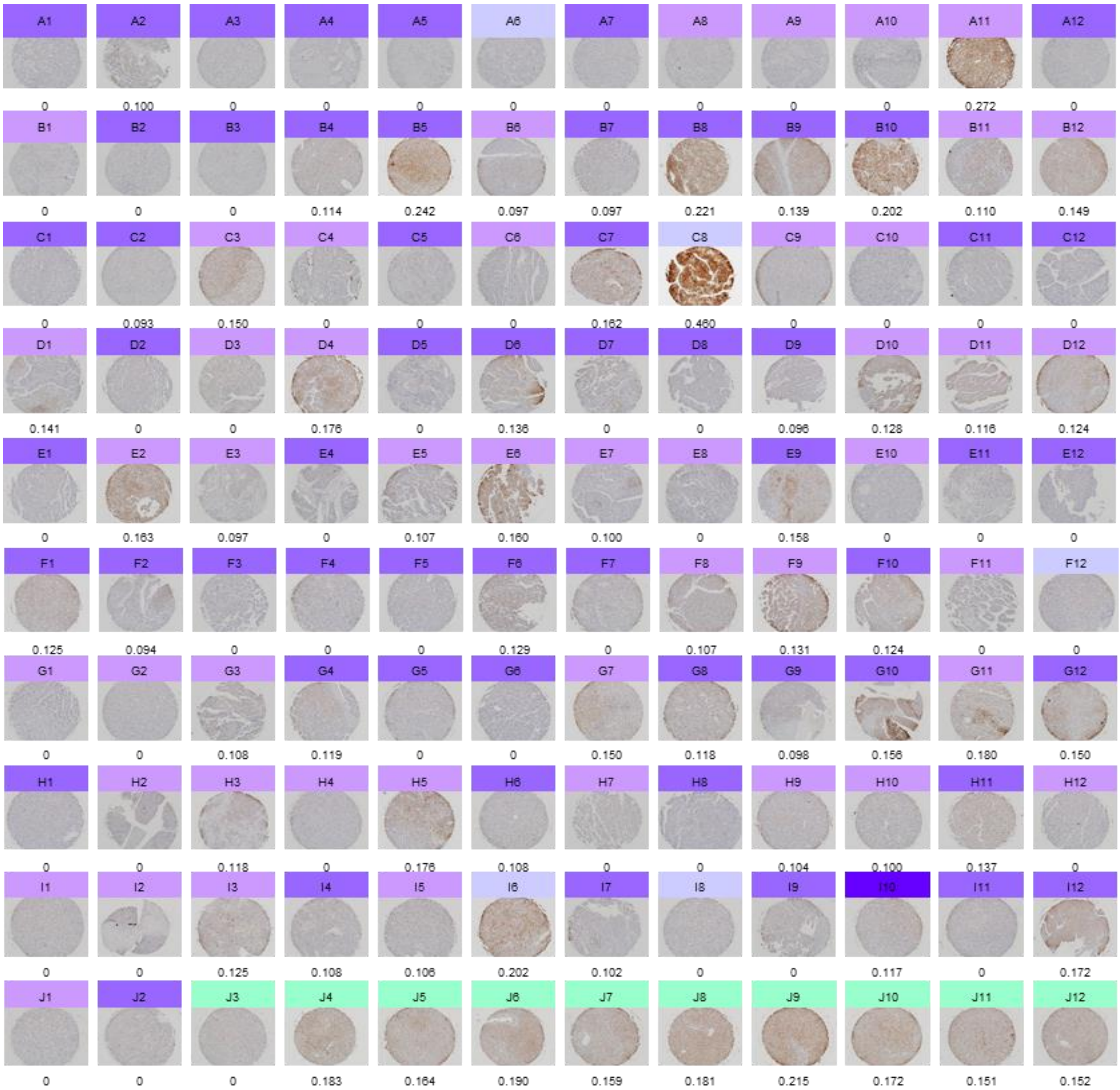

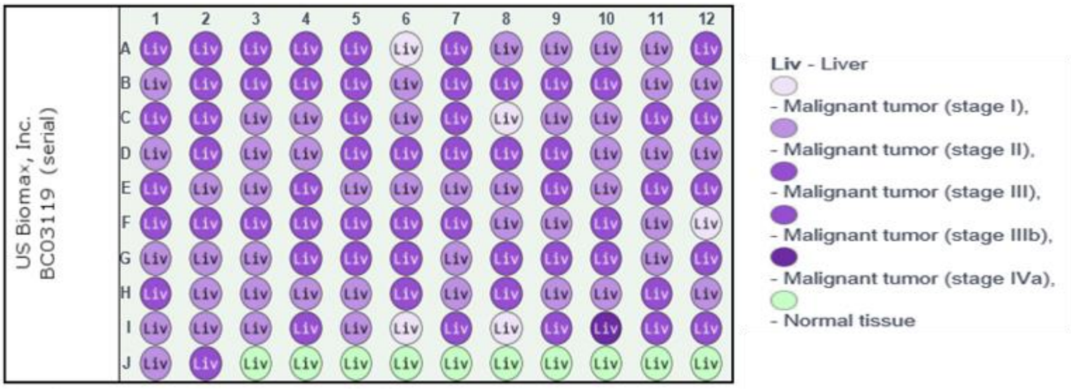

Supplementary figure S4. Immunohistochemical staining of human liver tissues microarray with anti-EIF3A antibody. The tissue microarray of liver cancer was purchased from US Biomax (cat no. BC03119) and stained with anti-EIF3A antibody (CST). The stained intensities of each sample were quantified with Image J and described under each image.

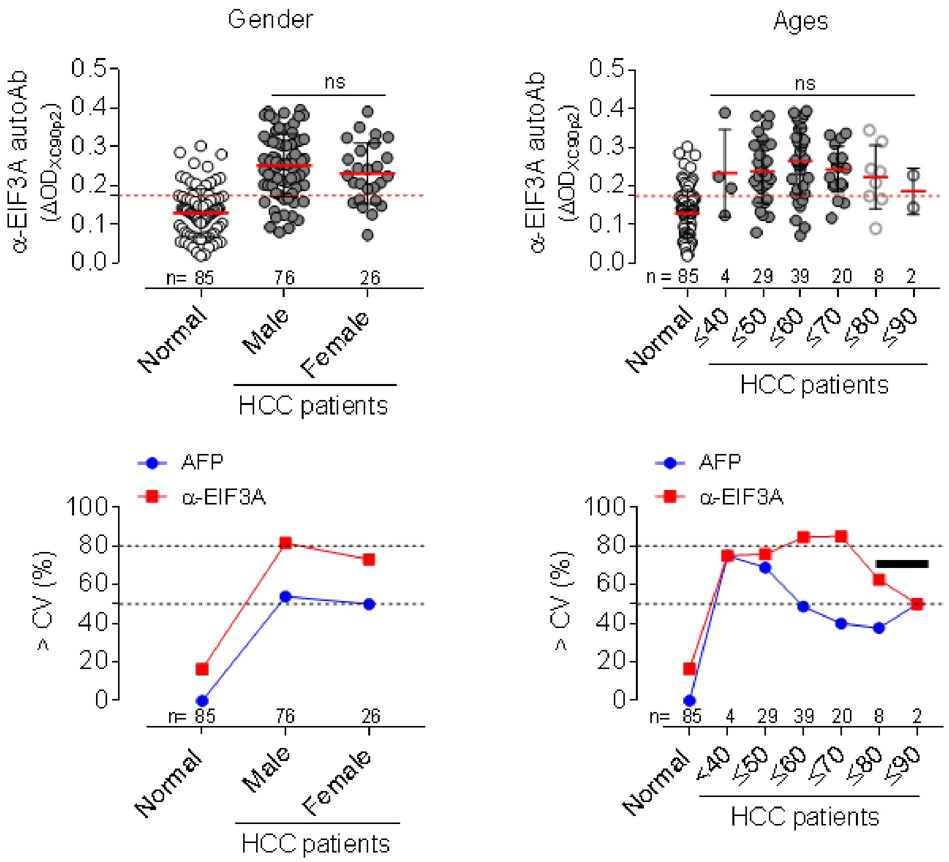

Supplementary figure S5. Anti-EIF3A autoantibody biomarker response of HCC patients depending on gender or age. The upper panel shows the anti-XC90p2 antibody response (ΔODXC90p2) of each serum sample. The lower panel shows the percentage of each group over the cutoff value (CV) of anti-EIF3A autoantibody test or AFP test. Anti-EIF3A autoantibody responses were above cutoff value in most of the subgroups of HCC patients (about 80%); however, elderly patients with HCC over 80 years showed a decrease in autoantibody response, which may be due to aging of the immune cells.

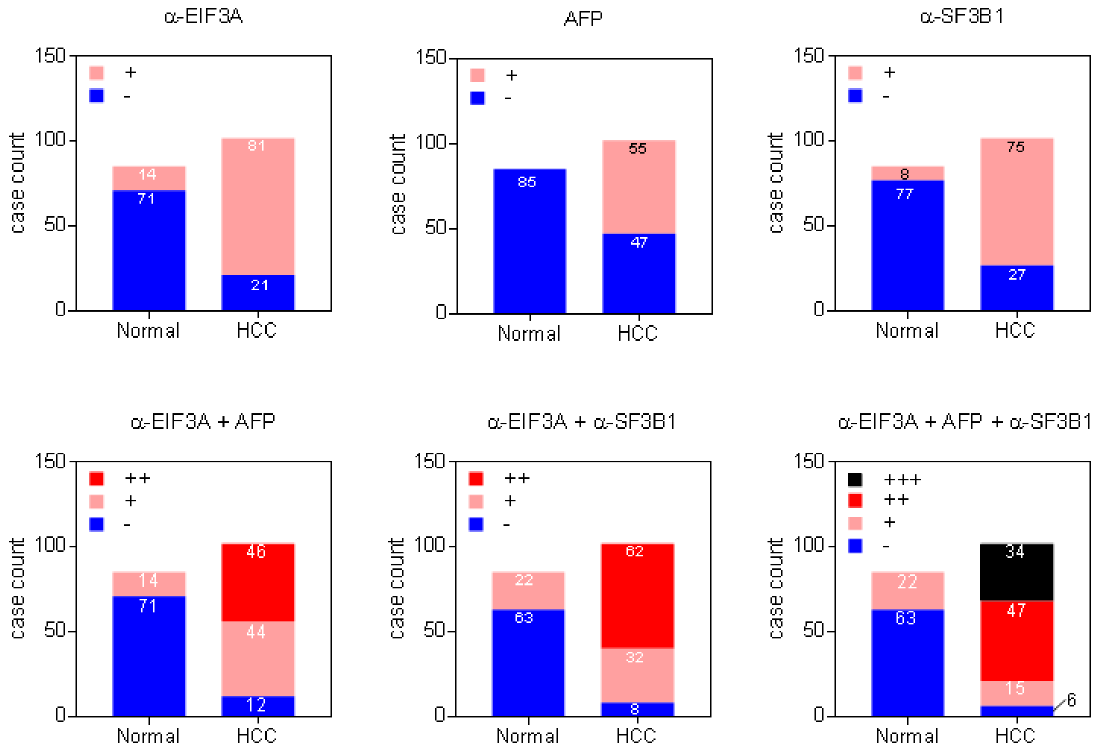

Supplementary figure S6. Analysis of biomarker tests with unified index. The diagnostic values of biomarkers (anti-EIF3A autoantibody, AFP, or anti-SF3B1 autoantibody) shown in Fig 4A and Fig 5A were simplified as either responsive (+) or non-responsive (-) according to whether their detection values were above or below the cutoff value and plotted (upper panels). Then, we analyzed the diagnostic values of their combination: for the combined analysis of these markers, we added the unified diagnostic indexes for a serum sample and designated triple negative as -, single positive as +, double positive as ++, and triple positive as +++. Numbers on plots represent the number of corresponding subjects.
